# Melatonin alleviates heat-induced damage of tomato seedlings by balancing redox homeostasis and modulating polyamine and nitric oxide biosynthesis

**DOI:** 10.1186/s12870-019-1992-7

**Published:** 2019-10-07

**Authors:** Mohammad Shah Jahan, Sheng Shu, Yu Wang, Zheng Chen, Mingming He, Meiqi Tao, Jin Sun, Shirong Guo

**Affiliations:** 1 Key Laboratory of Southern Vegetable Crop Genetic Improvement in Ministry of Agriculture, College of Horticulture, Nanjing Agricultural University, Nanjing, 210095 People’s Republic of China; 2 Department of Horticulture, Faculty of Agriculture, Sher-e-Bangla Agricultural University, Dhaka, 1207 Bangladesh

**Keywords:** Melatonin, Heat stress, Polyamines, NO biosynthesis, Redox, AsA-GSH cycle, Tomato

## Abstract

**Background:**

Melatonin is a pleiotropic signaling molecule that plays multifarious roles in plants stress tolerance. The polyamine (PAs) metabolic pathway has been suggested to eliminate the effects of environmental stresses. However, the underlying mechanism of how melatonin and PAs function together under heat stress largely remains unknown. In this study, we investigated the potential role of melatonin in regulating PAs and nitric oxide (NO) biosynthesis, and counterbalancing oxidative damage induced by heat stress in tomato seedlings.

**Results:**

Heat stress enhanced the overproduction of reactive oxygen species (ROS) and damaged inherent defense system, thus reduced plant growth. However, pretreatment with 100 μM melatonin (7 days) followed by exposure to heat stress (24 h) effectively reduced the oxidative stress by controlling the overaccumulation of superoxide (O_2_^•−^) and hydrogen peroxide (H_2_O_2_), lowering the lipid peroxidation content (as inferred based on malondialdehyde content) and less membrane injury index (MII). This was associated with increased the enzymatic and non-enzymatic antioxidants activities by regulating their related gene expression and modulating the ascorbate–glutathione cycle. The presence of melatonin induced respiratory burst oxidase (*RBOH*), heat shock transcription factors A2 (*HsfA2*), heat shock protein 90 (*HSP90*), and delta 1-pyrroline-5-carboxylate synthetase (*P5CS*) gene expression, which helped detoxify excess ROS via the hydrogen peroxide-mediated signaling pathway. In addition, heat stress boosted the endogenous levels of putrescine, spermidine and spermine, and increased the PAs contents, indicating higher metabolic gene expression. Moreover, melatonin-pretreated seedlings had further increased PAs levels and upregulated transcript abundance, which coincided with suppression of catabolic-related genes expression. Under heat stress, exogenous melatonin increased endogenous NO content along with nitrate reductase- and NO synthase-related activities, and expression of their related genes were also elevated.

**Conclusions:**

Melatonin pretreatment positively increased the heat tolerance of tomato seedlings by improving their antioxidant defense mechanism, inducing ascorbate–glutathione cycle, and reprogramming the PAs metabolic and NO biosynthesis pathways. These attributes facilitated the scavenging of excess ROS and increased stability of the cellular membrane, which mitigated heat-induced oxidative stress.

**Electronic supplementary material:**

The online version of this article (10.1186/s12870-019-1992-7) contains supplementary material, which is available to authorized users.

## Background

Global warming has led to climate change, including heat stress and these changes considerate as a major threat for worldwide crop production [1]. Heat stress can cause misfolds or disorganized cellular homeostasis because of excess reactive oxygen species (ROS) accumulation, distorted protein structure, impeded protein synthesis, and overall cell division and growth disruption from reduced water content [2–4]. Heat shock-induced oxidative damage occurs as a result of excess formation of singlet oxygen (_1_O^2^), superoxide radical (O_2_^•−^), hydrogen peroxide (H_2_O_2_), and hydroxyl radical (OH^•^) under heat stress [5]; this leads to overproduction of malondialdehyde (MDA) and thus reduces membrane stability, permeability, and mobility, and impairs protein membrane polymerization [6, 7]. As a sessile organism, in an unfavorable environment, plants develop an inherent antioxidative defense strategy to detoxify excess ROS, which helps to protect them from oxidative damage [8]. This efficient anti-oxidative defense mechanism consists of different enzymatic antioxidants, such as superoxide dismutase (SOD), catalase (CAT), peroxidase (POD), ascorbate peroxidase (APX), glutathione reductase (GR), monodehydroascorbate reductase (MDHAR), and dehydroascorbate (DHAR), and non-enzymatic antioxidants, such as ascorbate (AsA), glutathione (GSH), carotenoids, and phenols [2, 9, 10]. Additionally, heat-shock proteins (HSPs) play vital roles in ROS scavenging [11], because heat stress induces APX and CAT production [12]. Moreover, heat shock transcription factors A2 (*HsfA2*) plays a key role in the regulation of expression of heat-shock proteins, ascorbate peroxidase 2 and galactinol synthase 1 and 2 under high-temperature challenged [13].

Melatonin (N-acetyl-5-methoxytryptamine) is a naturally occurring low-molecular-weight multi-regulatory molecule that exists in all living organisms, including plants and animals [14, 15]. Since its detection in plants, scientists’ curiosity regarding melatonin has increased, because of its diversified biological role as a plant master regulator and defensive roles in capricious environmental conditions, such as extreme temperatures, salinity, drought, heavy metals, UV radiation, and oxidative stress [15–18]. Melatonin also accelerates seed germination [19], influences root and plant architecture [20], enhances growth vitality, ameliorates leaf senescence [21], regulates nitrogen metabolism [21], and alters physiological processes by inducing differential gene expression [16]. The most important function of melatonin is ROS detoxification through the production of free radicle scavengers (H_2_O_2_, O_2_^•−^) and modulation of both antioxidant enzyme activity and concentration [22, 23]. Rodriguez et al. [24] reported that pre-treated melatonin protects oxidative damage in cucumber through melatonin-mediated redox signaling pathways. Under both dark and light conditions, melatonin increases APX and CAT activity, and elevates AsA and GSH content; the AsA–GSH cycle also helps to reduce the dark-induced senescence [12, 25]. There is lack of research on how melatonin protects seedlings from possible damage caused by thermal stress. Consequently, the aim of this research was to elucidate melatonin’s mode of action.

Polyamines (PAs) are essential stress response biomolecules; they are small molecular weight nitrogenous compounds that exist ubiquitously in plants, mostly as putrescine (Put), spermidine (Spd), and spermine (Spm) [26]. PAs possess a wide variety of functions, including plant morphogenesis, reproductive stimulation, and delayed leaf senescence, and they play key roles against abiotic stresses, such as extreme temperature (high and low), salt, drought, heavy metals, osmotic stress, ultraviolet radiation stress, and submerged stress [27, 28]. Additionally, PAs have a cationic charge that helps uphold membrane integrity, assists smooth enzyme function, and protects DNA, RNA, and protein structure. Therefore, plant physiological, biochemical, and molecular activities are enhanced through interactions with nucleic acids, proteins, and phospholipids [29]. Ke et al. [28] determined that supplemental melatonin alleviates salinity stress in wheat seedlings by regulating PAs metabolism. Melatonin increases iron-deficiency tolerance through increased accumulation of PA-mediated nitric oxide (NO) [30]. Shi et al. [31] noted that the PAs metabolic system also changed under oxidative stress conditions with melatonin pretreatment in Bermuda grass. Moreover, melatonin increased Put and Spd levels in carrot suspension cells, which helped reduce cold-induce apoptosis [32]. Additionally, melatonin pretreatment alleviated chilling stress in harvested peach fruits [33] and cucumber seedlings [34] which are closely related to PAs metabolism. These research findings revealed that melatonin may play critical roles in capricious environments by mediating PAs metabolism. Alternatively, the signaling molecule NO functions as a mediator of PAs metabolism and plant hormones, and also triggers NO biosynthesis [35]. However, until now, how melatonin regulates PAs metabolism have not been entirely understood. We hypothesize that melatonin may be associated with PAs via the NO biosynthesis pathway, thus helping plants cope with high-temperature challenges. Correlations between melatonin biosynthesis and PAs metabolism, and their underlying mechanisms could provide a novel insight that can help to promote plant production and protection.

## Result

### Melatonin improved morphological parameters in tomato seedling under heat stress

Fresh weight (FW) and dry weight (DW) of shoots and roots significantly decreased in high-temperature treatment seedlings, especially root FW and DW (Table 1). Shoot and root FW were reduced by around 30 and 12%, respectively, under heat stressed seedlings compared to normal growth conditions. Conversely, exogenous melatonin mitigated temperature-induced inhibition of growth components and facilitated better growth.

**Table 1:** Effects of melatonin on the morphology of heat stress exposed tomato seedlings

Treatments	Shoot fresh weight (g plant^−1^)	Shoot dry weight (g plant^− 1^)	Root fresh weight (g plant^− 1^)	Root dry weight (g plant^− 1^)
CK	8.65 ± 0.17a	0.88 ± 0.05a	2.30 ± 0.26b	0.16 ± 0.01ab
MT	8.90 ± 0.15a	0.91 ± 0.06a	4.17 ± 0.13a	0.21 ± 0.02a
HT	6.03 ± 0.18b	0.47 ± 0.02c	2.04 ± 0.15b	0.12 ± 0.05b
MT + HT	8.03 ± 0.16a	0.65 ± 0.03b	4.04 ± 0.21a	0.20 ± 0.02a

Data represent as a mean of standard deviation (SD) of three replications. Different letters indicate significant differences according to Tukey’s HSD test at *P* ≤ 0.05

CK: Control, MT: 100 μM melatonin, HT: High temperature (42 °C), MT + HT: 100 μM melatonin + high temperature (42 °C)

### Melatonin controlled the overaccumulation of ROS in heat stressed tomato seedlings

**Fig. 1:**
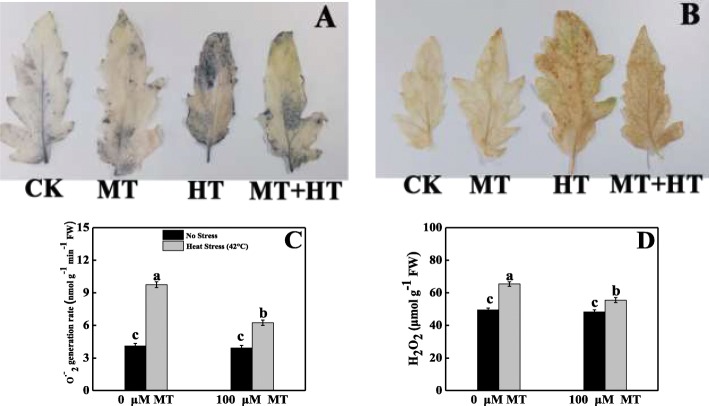
Effects of Melatonin (100 μM) on accumulation of (**A**) superoxide ion (O_2_^•−^), (**B**) hydrogen peroxide (H_2_O_2_), (**C**) generation of superoxide ion (O_2_^•−^) and (**D**) content of hydrogen peroxide (H_2_O_2_) in leaves of tomato seedlings in presence or absence of high temperature (42 °C) stress. CK: Control; MT: 100 μM melatonin; HT: High temperature (42 °C); MT + HT: 100 μM melatonin + high temperature (42 °C). Data represent as a mean of standard deviation (SD) of three replications. Different letters indicate significant differences according to Tukey’s HSD test at *P* ≤ 0.05

To investigate if melatonin alleviates heat stress-induced oxidative stress, we first detected H_2_O_2_ and O_2_^•−^ generation in tomato leaves by histochemical staining. As shown in Fig. 1a, b, we observed deeper blue staining, which indicates O_2_^•−^ production, and acute brown staining, which denotes H_2_O_2_ production, on the leaf surface of high temperature-stressed tomato seedlings. Conversely, the differences among the other treated seedlings were less in their leaf blades, which indicates that melatonin inhibits ROS overproduction under stress conditions.

To evaluate the ROS accumulation trends under melatonin and/or heat stress, we further examined the generation rates of O_2_^•−^ and H_2_O_2_ in tomato leaves (Fig. 1c, d). Relative to the control, sharp increases in O_2_^•−^ and H_2_O_2_ production were observed in the leaf tissues upon subjected to heat stress. Under heat stress, H_2_O_2_ and O_2_^•−^ contents increased by 32 and 137%, respectively, compared with the control seedlings. By contrast, application of melatonin increased the heat-stress tolerance of seedlings by reducing the formation rates of H_2_O_2_ and O_2_^•−^ in leaf tissue by 15 and 36%, respectively, compared with the plants grown solely in the high-temperature environment.

### Melatonin maintained cellular membrane integrity in tomato leaves under heat stress

**Fig. 2:**
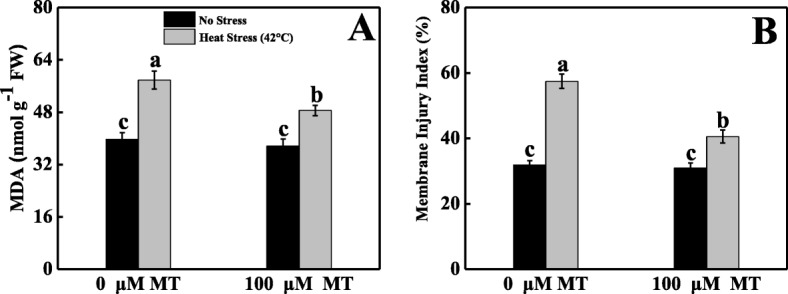
Effects of Melatonin (100 μM) on (**A**) malondialdehyde (MDA) content and (**B**) membrane injury index (MII) in leaves of tomato seedlings in presence or absence of high temperature (42 °C) stress. Data represent as a mean of standard deviation (SD) of three replications. Different letters indicate significant differences according to Tukey’s HSD test at *P* ≤ 0.05

High temperatures destroyed tomato seedling leaves cellular membranes as indicated by MII (80%) and the higher accumulation of MDA content (45%) compared with the control (Fig. 2). Application of 100 μM melatonin was more effective for overcoming harsh impacts of heat stress, as shown by a substantial reduction of MII (29%) and lower MDA concentration (16%) compared with untreated heat-stressed plants.

### Melatonin enhanced proline metabolism and RWC in tomato seedlings under heat stress

**Fig. 3:**
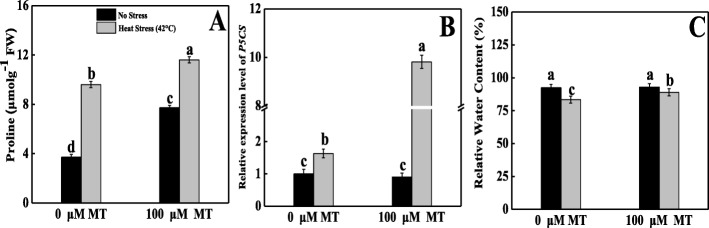
Effects of Melatonin (100 μM) on (**A**) proline content and (**B**) relative transcript expression of *P5CS* and (**C**) relative water content (REL) in leaves of tomato seedlings in presence or absence of high temperature (42 °C) stress. Data represent as a mean of standard deviation (SD) of three replications. Different letters indicate significant differences according to Tukey’s HSD test at *P* ≤ 0.05

Heat-stressed plants induced elevated proline biosynthesis that was 158% greater compared with the corresponding control (Fig. 3a). The melatonin-pretreatment combined with heat-stressed seedlings showed a maximum proline content that was 212% greater than that of control. To verify this, we further quantified the gene expression of *P5CS*, which is responsible for proline biosynthesis. The *P5CS* expression pattern was substantially upregulated (1.62-fold) in heat-stressed seedlings compared with the control plants. Melatonin pretreatment in heat-stressed seedlings further markedly upregulated *P5CS* expression by 6-folds in contrast with seedlings that were grown only heat-stressed conditions (Fig. 3b).

For RWC, in respect to normal plants, high-temperature challenge seedlings considerably decreased the RWC by 10%; supplemental melatonin application curtailed a significant amount of water loss from their tissues, and these plants contained 7% more RWC than the untreated heat-stressed seedlings (Fig. 3c).

### Melatonin balanced the antioxidant defense system in tomato seedlings under heat stress

**Fig. 4:**
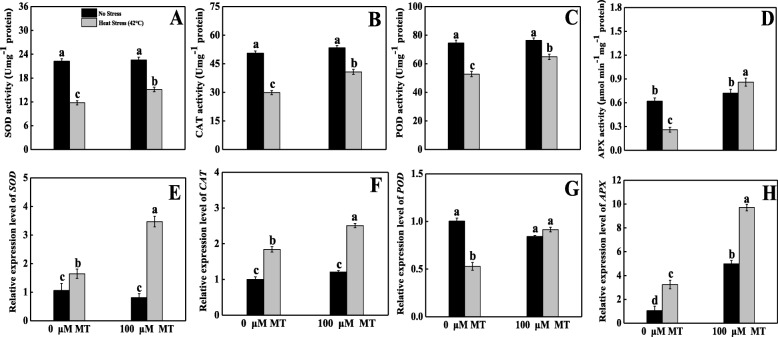
Effects of Melatonin (100 μM) on antioxidant enzymes activities and their related transcript expression of (**A, E**) superoxide dismutase (SOD), (**B, F**) catalase (CAT), (**C, G**) peroxidase (POD) and (**D, H**) ascorbate peroxidase (APX) in leaves of tomato seedlings in presence or absence of high temperature (42 °C) stress. Data represent as a mean of standard deviation (SD) of three replications. Different letters indicate significant differences according to Tukey’s HSD test at *P* ≤ 0.05

To evaluate the role of melatonin in oxidative stress remediation, we investigated the activities of antioxidant enzymes upon exposure to heat stress (Fig. 4). Under heat stress, SOD activity substantially declined and was 1.89-fold lower than in the control; alternatively, SOD activity significantly increased and was 1.29-fold higher in plants with melatonin pretreatment under high temperatures than untreated heat-stressed seedlings (Fig. 4a). Heat stress caused a marked decrease of 41% in CAT activity in leaves compared with normally grown leaves; melatonin-pretreated heat-stressed seedlings had upwards of 36% greater in CAT activity than untreated heat-stressed leaves (Fig. 4b). Melatonin-pretreated heat-stressed seedlings had dramatically increased POD activity (by 23%) in contrast with untreated heat-stressed seedlings; however, relative to control plants, POD activity sharply declined in untreated heat-stressed seedlings (29%, Fig. 4c). Under heat stress, APX activity decreased by 60% compared with the control, whereas, melatonin pretreatment in heat-stressed seedlings resulted in 230% greater APX activity than that of untreated heat-stressed seedlings (Fig. 4d).

### Melatonin induced the AsA–GSH cycle and homeostasis in tomato seedlings under heat stress

**Fig. 5:**
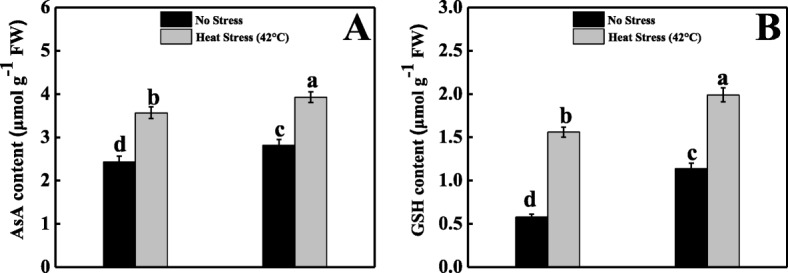
Effects of Melatonin (100 μM) on (**A**) ascorbic acid (AsA) and (**B**) glutathione (GSH) content in leaves of tomato seedlings in presence or absence of high temperature (42 °C) stress. Data represent as a mean of standard deviation (SD) of three replications. Different letters indicate significant differences according to Tukey’s HSD test at *P* ≤ 0.05

As displayed in Fig. 5a, AsA content was markedly increased by 47% after heat stress in contrast to the control plants. Moreover, in response to normal seedlings, AsA content was further increased by 62% in melatonin-pretreated heat-stressed seedlings. Under exposure to heat stress, the GSH content was remarkably increased (168%) in contrast to the corresponding control seedlings (Fig. 5b). Alternatively, melatonin-pretreated heat-stressed seedlings had 28% greater GSH content with respect to the untreated heat-stressed seedlings.

**Fig. 6:**
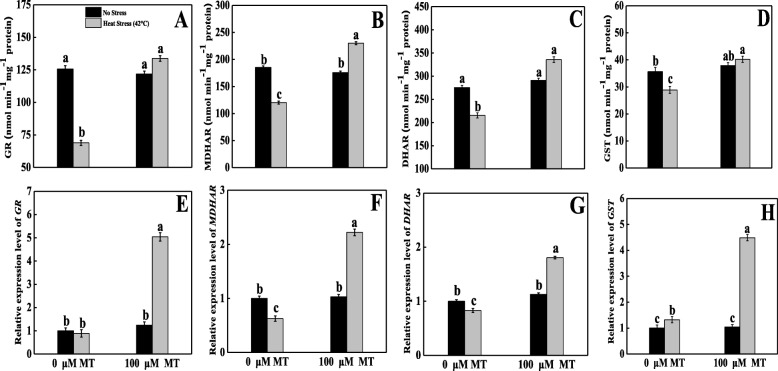
Effects of Melatonin (100 μM) on antioxidant enzymes activities and their related transcript expression of (**A, E**) glutathione reductase (GR), (**B, F**) monodehydroascorbate reductase (MDHAR), (**C, G**) dehydroascorbate reductase (DHAR) and (**D, H**) glutathione S-transferase (GST) in leaves of tomato seedlings in presence or absence of high temperature (42 °C) stress. Data represent as a mean of standard deviation (SD) of three replications. Different letters indicate significant differences according to Tukey’s HSD test at *P* ≤ 0.05

There were no significant differences in GR activity among the treatments except in the untreated heat-stressed seedlings (Fig. 6a). However, GR activity sharply decreased by 45% in thermal-stressed plants with respect to the control seedlings. By contrast, when plants treated with melatonin followed by high-temperature exposure showed upregulation of GR activity by 94% than seedlings subjected to heat-stressed alone.

However, marked decreases of MDHAR and DHAR activities were observed in heat-stressed seedlings compared to other seedlings. Additionally, in respect to only heat-stressed seedlings MDHAR and DHAR enzyme contents rose by 91 and 56%, respectively, in melatonin-pretreated heat-stressed seedlings (Fig. 6b, c).

In untreated heat-stressed seedlings, the GST activity slightly decreased by around 19% compared with the control groups. In contrast, melatonin pretreatment increased GST activity by 39% than untreated heat-stressed seedlings (Fig. 6d).

### Melatonin modulated the transcription of enzymatic antioxidants under heat stress

To elucidate the molecular mechanism underlying how melatonin alleviates heat stress-induced oxidative damage, the transcript levels of some key genes that encode antioxidant enzymes were assayed. The results showed that SOD (Fig. 4e), CAT (Fig. 4f), APX (Fig. 4h), and GST (Fig. 6h) gene expression levels were upregulated by 1.6-, 1.2-, 3.2-, and 1.3-fold, respectively, in response to their corresponding control groups. Alternatively, the application of exogenous melatonin upregulated SOD, CAT, POD (Fig. 4g), APX, GR (Fig. 6e), MDHAR (Fig. 6f), DHAR (Fig. 6g), and GST expression by 2.1-, 1.3-, 1.7-, 3.0-, 5.6-, 2.4-, 1.8-, and 3.4-fold, respectively, compared with untreated heat-stressed seedlings.

### Melatonin regulated the *RBOH* expression in tomato leaves under heat stress

**Fig. 7:**
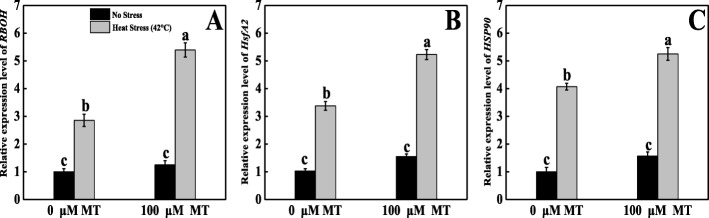
Effects of Melatonin (100 μM) on relative transcript expression of (**A**) Respiratory burst oxidase (*RBOH*) and (**B**) heat shock factor A 2 (*HsfA2*) (**C**) heat shock protein 90 (*HSP90*) in leaves of tomato seedlings in presence or absence of high temperature (42 °C) stress. Data represent as a mean of standard deviation (SD) of three replications. Different letters indicate significant differences according to Tukey’s HSD test at *P* ≤ 0.05

As indicated in Fig. 7a, we analyzed the tomato *RBOH* expression level. The *RBOH* transcript levels were prominently elevated in heat-stressed seedlings than control ones. Conversely, exogenously applied melatonin with subsequent high-temperature exposure also increased *RBOH* expression by 1.89-fold compared with untreated heat-stressed plants.

### Melatonin regulated the heat shock-related genes expression in tomato leaves under heat stress

As shown in Fig. 7b, c, *HSP90* and *HsfA2* expression levels were increased by 4.1- and 3.26-fold, respectively, in untreated heat-stressed seedlings compared with control seedlings. In contrast to untreated heat-stressed seedlings, the *HSP90* and *HsfA2* transcript levels were sharply upregulated by 1.29- and 1.55-fold, respectively, in melatonin-pretreated heat-stressed seedlings.

### Melatonin modulated endogenous levels of PAs and their genes expression in tomato leaves under heat stress

**Fig. 8:**
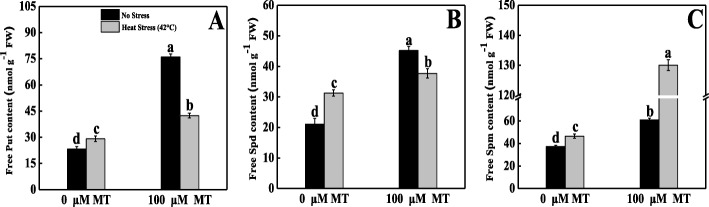
Effects of Melatonin (100 μM) on endogenous free polyamines content of (**A**) Putrescine (Put), (**B**) Spermidine (Spd), (**C**) Spermine (Spm) in leaves of tomato seedlings in presence or absence of high temperature (42 °C) stress. Data represent as a mean of standard deviation (SD) of three replications. Different letters indicate significant differences according to Tukey’s HSD test at *P* ≤ 0.05

We quantified endogenous free PAs accumulation to explicate how PAs and melatonin coordinate in order to eliminate the adverse effects of thermal stress. The Put, Spd, and Spm contents significantly increased in heat-stressed seedlings by 25.26, 48.24, and 24.43%, respectively, compared with the control group (Fig. 8). Melatonin supplementation further increased Put by 82.52%, Spd by 78.72%, and Spm by 247.80% relative to their corresponding control plants.

**Fig. 9:**
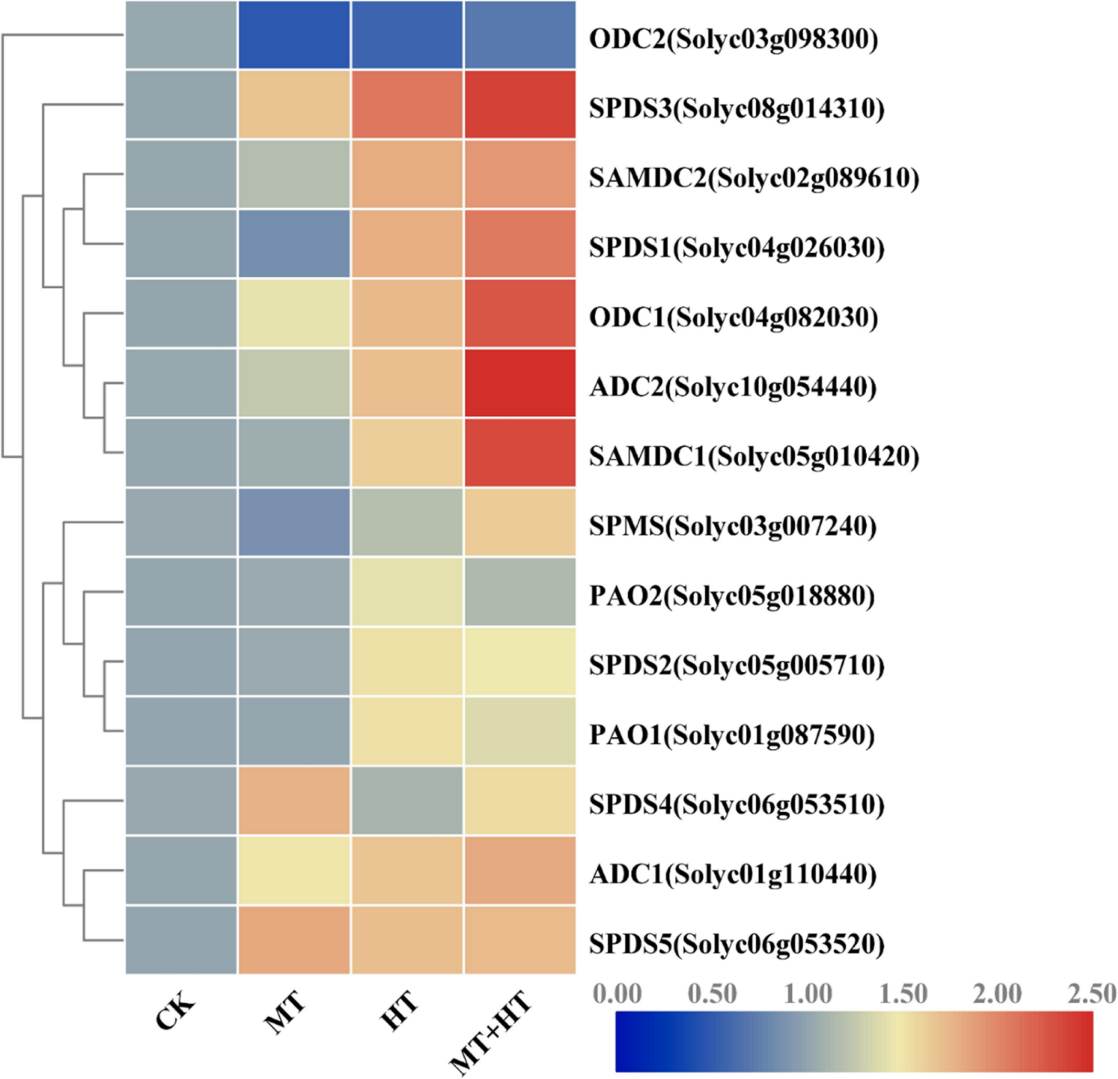
Heat-map representing the relative transcript abundance of differentially expressed Polyamines genes in leaves of tomato seedlings under high temperature stress with or without melatonin pretreatment and also hierarchical cluster analysis were used. The gene expression intensity extended from blue color (low) to red color (high). CK: Control; MT: 100 μM melatonin; HT: High temperature (42 °C); MT + HT: 100 μM melatonin + high temperature (42 °C)

To reveal the expression profile of PAs metabolism, we performed heatmap visualization and hierarchical cluster analysis (Fig. 9). In the presence of melatonin, the mRNA levels of PAs metabolic genes showed higher expression than control. High-temperature stress exposure with or without melatonin treatments caused upregulation of the transcript levels of the assayed genes. The *ADC1*, *ADC2*, and *ODC1* mRNA levels were increased in stressed seedlings, with and without melatonin, whereas *ODC2* expression was downregulated in all heat-stressed seedlings compared with control seedlings. Similarly, the *SAMDC1*, *SAMDC2*, and *SPMS* transcript abundance also increased in response to either all heat-stressed seedlings compared with control seedlings. Furthermore, in contrast to untreated heat-stressed seedlings, the *SPDS1*, *SPDS2*, *SPDS3, SPDS4,* and *SPDS5* expression levels were also upregulated in melatonin-pretreated plants. Interestingly, the *PAO1* and *PAO2* transcript levels were downregulated in melatonin-pretreated seedlings than untreated heat-stressed plants, which indicates that melatonin might inhibit heat-induced damage via the PAs metabolic pathway and not a catabolic process.

### Melatonin regulated the NO biosynthesis pathway under heat stress

**Fig. 10:**
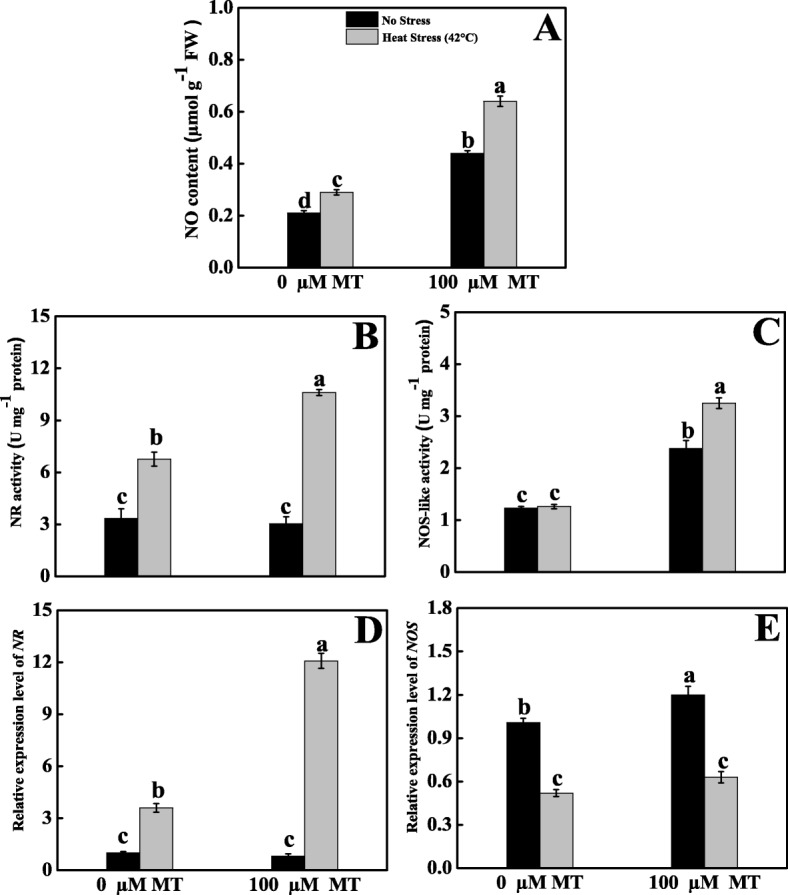
Effects of Melatonin (100 μM) on NO biosynthesis pathway in leaves of tomato seedlings in presence or absence of high temperature (42 °C) stress. **A** NO content, **B, D** NR activity and its transcript expression level, **C, E** NOS-like activity and its transcript level. Data represent as a mean of standard deviation (SD) of three replications. Different letters indicate significant differences according to Tukey’s HSD test at *P* ≤ 0.05

As displayed in Fig. 10, untreated heat-stressed plants released 38% more NO than control seedlings. In contrast, melatonin pretreatment further increased the NO content in thermal-stressed tomato seedlings by approximately 205% than control plants. NR activity increased upon exposure to high temperatures with or without melatonin applied to the seedlings. Melatonin-pretreated heat-stressed seedlings markedly increased NR activity in respect to control plants by around 218%.

Alternatively, in the case of NOS-like activity, there was not significant variation between control and heat-stressed seedlings. The NOS-like activity was dramatically increased by 164% in melatonin-pretreated heat-stressed seedlings in contrast with control seedlings. The relative transcript level of NR was increased (2.6- fold) in untreated heat-stressed seedlings than control seedlings. However, NR expression was further exponentially upregulated (2.35- fold) in melatonin-pretreated heat-stressed seedlings in respect to untreated heat-stressed seedlings. However, in the case of NOS, expression was downregulated in all heat-stressed seedlings with or without melatonin pretreatment compared with the control seedlings.

## Discussion

Melatonin is a pleiotropic molecule that is involved in diverse plant physiological functions, including seed morphogenesis, growth, and development; root architecture; photosynthesis; and chlorophyll pigment production; and it is a plant master regulator and defensive player in capricious environments [16]. Cellular oxidative damage is a stress marker of high-temperature stress, and H_2_O_2_ and O_2_^•−^ generation, MDA content, and MII are the representative of these stress markers. Heat-stressed tomato leaves conspicuously displayed deep blue spots, which indicated that they produced more O_2_^•−^. The leaves also had dark brown patches, which indicated greater accumulation of H_2_O_2_ under the same stress conditions. These staining symptoms were supported by quantification of H_2_O_2_, and O_2_^•−^, which showed markedly higher generation under elevated temperatures compared with melatonin-pretreated heat-stressed seedlings (Fig. 1). These findings are consistent with prior findings that melatonin decreased H_2_O_2_ and O_2_^•−^ accumulation in kiwifruit [36], watermelon [37], cucumber [38], and *Malus hupehensis* [39]. The probable mechanism underlying this decrease may be that melatonin acts as an electron donor [40]. MDA often represents an essential stress symptom that forms through an auto-oxidative chain reaction as a result of ROS-induced bio-membrane damage [41]. However, in a capricious environment, the MII and MDA concentration are predominantly associated with each other. Plants exposed to heat stress had sharply increased MDA levels that could potentially damage the plasma membrane integrity, which elevated MII in tomato seedlings (Fig. 2). Melatonin application decreased both MDA and MII, which is consistent with the findings of previous studies on kiwifruit [36, 42], Bermuda grass [31], and tomato [43] under various abiotic stresses. These results indicate that melatonin may be able to repair the disrupted cellular membrane and reduce heat-induced oxidative damage by balancing ROS in a high-temperature environment. *HsfA2* and *HSP90* are the key regulators that stimulate ROS detoxification through the H_2_O_2_-mediated signaling pathway and, therefore, increase plant thermo-tolerance. In this study, melatonin-pretreated tomato seedlings had upregulated *HsfA2* and *HSP90* expression compared with untreated heat-stressed seedlings (Fig. 7), which indicates that melatonin ameliorated the heat stress-induced oxidative damage caused by *HsfA2* and *HSP90* activation. A recent report also revealed that *HsfA2* plays roles in H_2_O_2_ signaling and increases heat stress memory subsistence, whereas *HSP90* coordinates DNA-binding enhancement process and HSF balanced in plants exposed to heat stress, and this whole mechanism might be related to melatonin-mediated heat tolerance [22, 44–46].

Melatonin is a dynamic antioxidant [47, 48] that extensively stimulates cellular redox homeostasis by enhancing the activity of enzymatic antioxidants, including SOD, CAT, POD, APX, GR, MDAR, and DHAR, and non-enzymatic antioxidants, including AsA and GSH [49–52]. Therefore, melatonin helps detoxify excess ROS, which helps plants survive under stressful conditions. We assayed enzyme activity and conducted expression analysis of antioxidant-related genes and observed that all the antioxidant-related enzymes activities were reduced under thermal stress. Conversely, melatonin-pretreated heat-stressed plants showed higher SOD, CAT, and POD activity relative to untreated heat-stressed plants (Fig. 4). The first mechanism of defense against ROS in plants is through SOD, which eliminates O_2_^•−^ by converting it into O_2_ and H_2_O_2_ [53]. In addition, CAT and POD also actively participate in scavenging H_2_O_2_, which they convert into H_2_O and O_2_ [54], which indicates that these enzymes have a vital role in scavenging more H_2_O_2_, and these and the MDA results were consistent with those of melatonin-treated kiwifruit [42], wheat [55], and tea [17].

Moreover, we speculated that heat stress elevated the proline content, upregulated the proline biosynthesis gene (*P5CS*), and lowered the water content in leaves, whereas melatonin pretreatment further increased the proline level, *P5CS* expression, and leaf water content. This finding indicates that melatonin has the potential to help leaves maintain a higher water level and lower cellular osmotic potential (Fig. 3) by the proline biosynthesis pathway, which enhanced the plants’ ability to cope with heat stress [56]; this was also supported by previous experimental results [57].

Alternatively, ascorbate is a vital antioxidant enzyme that substantially detoxifies ROS; APX and GR are also crucial enzymes in the AsA–GSH cycle. The activities of APX, MDHAR, DHAR, and GR enzymes were only decreased in heat stress-exposed seedlings, whereas melatonin pretreatment elevated the AsA content as a result of increased the activities of APX, MDHAR, DHAR, and GR enzymes under heat stress. All of these enzymes actively contributed to the AsA–GSH cycle, which converts the tiny non-enzymatic molecules AsA and GSH [58]. The AsA values depend upon metabolizing (APX) and recycling (MDHAR, DHAR) enzyme activity. Moreover, at the time of ROS detoxification, DHAR oxidizes GSH to GSSG. Simultaneously, GR recycles GSH. Therefore, we concluded that melatonin pretreatment might have the potential to reduce oxidative damage by inducing the AsA–GSH cycle [59, 60]. To elucidate the inherent mechanisms, we quantified the expression of several related genes. Under heat stress, *RBOH* expression was upregulated and the expression level was further magnified in melatonin-pretreated heat-stressed plants (Fig. 7a). In melatonin-pretreated heat-stressed seedlings, the relative transcript abundance of enzymatic (SOD, CAT, POD) and non-enzymatic (APX, GR, MDHAR, DHAR, GST) antioxidant genes were upregulated (Figs. 4 and 6), which indicates that plants were more stable under heat stress because of excess ROS scavenging; these findings are consistent with those of previous research done on kiwifruit [42], watermelon [52], apple [61], and *Arabidopsis* [62] under various abiotic stress conditions.

PAs play a critical role in plant signaling transduction that is beneficial for counteracting the effects of different capricious environments [27]. Some previous studies determined that melatonin has a positive regulatory effects on plant development and abiotic stress (alkaline stress, cold, thermal, oxidative, and iron deficiency tolerance) management by interacting with the PAs signaling pathway [28, 63]. Melatonin might ameliorate the thermal oxidative stress by interacting with the PAs and NO biosynthesis pathways. The exogenous application of melatonin elevated the endogenous free PAs level. Similarly, expression levels of different PAs biosynthesis genes were also upregulated in melatonin-pretreated heat-stressed seedlings. The transcript abundances of *ADC1/2*, *SAMDC1/2, SPMS,* and *SPDS1/2/3/5/6* were upregulated (Fig. 9), and that of *PAO1/2* was downregulated in melatonin-pretreated heat-stressed seedlings, and these genes are associated with Put, Spd, and Spm biosynthesis. These findings indicate that melatonin and PAs metabolism have close interactions. This finding is also similar to that of previous research performed on various crops under different stresses [30, 32–34, 64]. Alam et al. [65] concluded that long-term heat-stressed seedlings treated with melatonin adjusted through the modulation of PAs metabolism.

Melatonin along with NO has the potential to combat different stress conditions through the L-arginine and PAs metabolic pathways [66]. However, the NOS and NR pathways are also regulated via PAs [67, 68]. Our current data also highlight that the NO content, NR activity, and NOS-like activity along with the expression of their related genes were elevated in melatonin-pretreated heat-stressed tomato seedlings (Fig. 10), which indicates that melatonin triggered the NO activity [30]. Overall, melatonin enhanced mitigation of heat-induced damage through coordination with PA- and NO-mediated signaling pathways.

## Conclusions

**Fig. 11:**
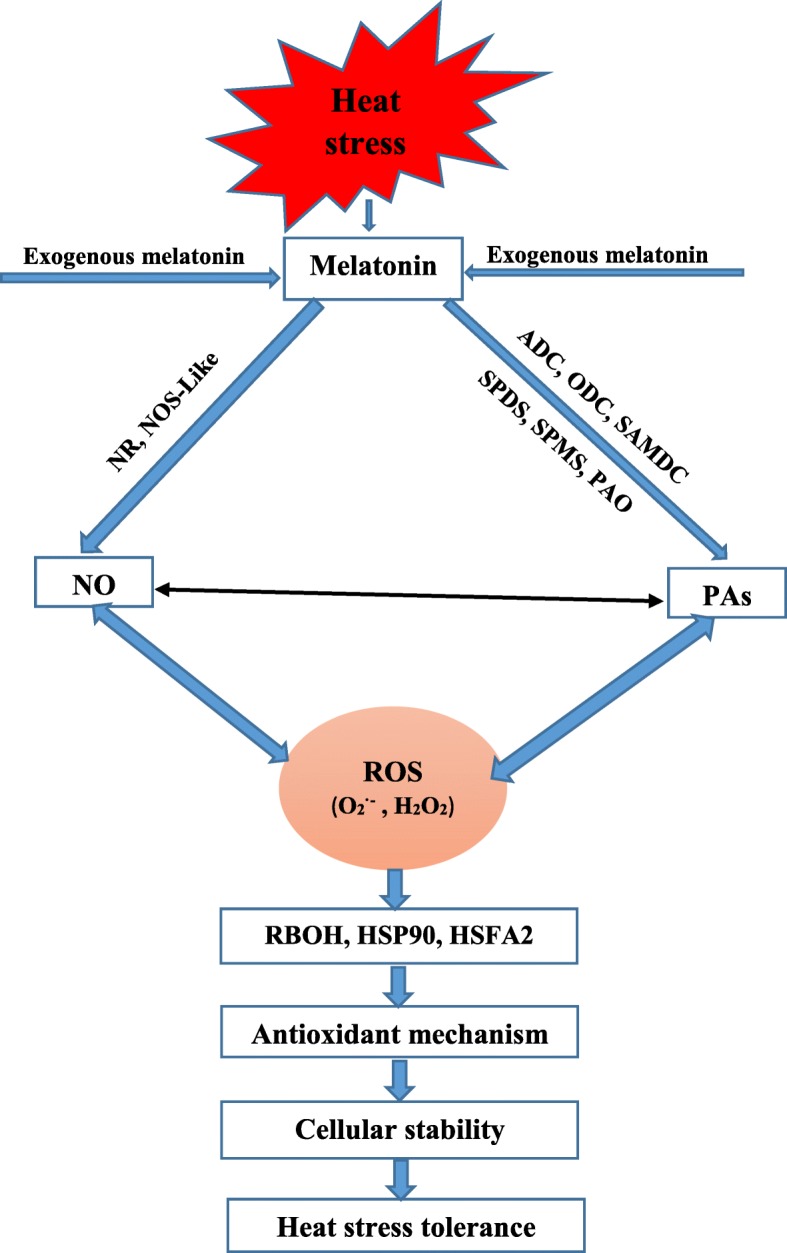
The schematic representation of possible mechanism of melatonin mediated high temperature stress tolerance in tomato seedlings via the PAs and NO biosynthesis pathway. PAs: polyamines; ADC: arginine decarboxylase; ODC: ornithine decarboxylase; SPDS: spermidine synthase; SPMS: spermine synthase; SAMDC: s-adenosyl methionine decarboxylase; PAO: polyamine oxidase; NO: Nitric oxide; NR: nitrate reductase; NOS: nitric oxide synthase; ROS: reactive oxygen species; H_2_O_2_: hydrogen peroxide; O_2_^•−^: superoxide anion; RBOH: Respiratory burst oxidase; HSFA2: heat shock transcription factors A2; HSP90: heat shock protein 90

To determine how melatonin mitigated heat stress-induced adverse effects in tomato seedlings, we described a probable mechanism (Fig. 11). We observed that 100 μM exogenous melatonin treatment improved the thermal tolerance of tomato seedlings by lowering ROS (H_2_O_2_, O_2_^•−^, MDA) production, enhanced antioxidant enzyme activity, AsA–GSH cycle modulation, and upregulation of antioxidant-related gene expression. Additionally, melatonin elevates endogenous PAs via upregulation of PAs biosynthesis genes. NO content along with NR and NOS activity were also increased with melatonin supplementation. Therefore, we concluded that heat stress-induced damage was suppressed by melatonin, which coordinates with the PAs and NO biosynthesis pathways, which helps to detoxify the overaccumulated ROS. These findings provide novel insight into the cross-talk that exists among melatonin, PAs, and NO to inhibit thermal stress. To better understand this phenomenon, further investigation is needed to determine how these three molecules collectively function to alleviate the heat-stress induced damage.

## Methods

### Plant material and growth conditions

Tomato (*Solanum lycopersicum* L. Cv. Hezuo 903) seeds (Shanghai Tomato Research Institute, Shanghai, China) were sorted by uniform size and then sterilized by 0.1% sodium hypochloride (NaOCl) for 5 min, followed by washing several times with deionized water; then, they were placed in dark conditions for 36 h at 28 ± 1 °C for germination. Germinated seeds were then sown in plastic trays (41 × 41 × 5 cm) that contained a peat and vermiculite (2:1, v:v) mixture and cultured in a growth chamber at Nanjing Agriculture University, where the environmental conditions were maintained at 28 ± 1 °C (day) and 19 ± 1 °C (night), relative humidity from 65 to 75%, and 12 h photoperiods (PAR 300 μmol m^− 2^ s^− 1^). After the second true leaf was fully expanded, the uniformly grown seedlings were selected and transferred into containers filled with a peat and vermiculite (2,1, v, v) mixture and watered on alternate days with full-strength Hoagland solution.

### Treatment and sampling

When the fourth true leaves were fully developed, the seedlings were divided into two sub-groups for challenge under different treatments. Melatonin was applied as described in previous experiment performed by Martinez et al. [69]. In the first sub-group, 80 mL of 100 μM melatonin was sprayed on each tomato seedling leaves each day and for 7 days; in the second sub-group, each tomato seedling leaves were sprayed with the same volume of water. Melatonin stock solution was prepared by dissolving melatonin in ddH_2_O with 0.01% v/v Tween-20 used as a surfactant. One week after treatment, half of the melatonin-treated seedlings and half of the water-sprayed seedlings were separated and exposed to a high temperature (42 °C) for 24 h [10]. After 24 h of heat treatment, leaves were harvested for subsequent analysis and immediately stored at − 80 °C.

### Measurement of growth indicators

To assess the combined effects of melatonin and heat-stressed in tomato seedlings, we measured different growth indicators such as fresh and dry weight of leaves and roots. Fresh weight of leaves and roots were measured by electric balance. For dry weight records plants were oven dried (80 °C for 72 h).

### Histochemical detection of H_2_O_2_ and O_2_^•−^

H_2_O_2_ and O_2_^•−^ generation rate were detected using 3,3- diamino benzidine (DAB) and nitro blue tetrazolium (NBT), respectively, using a previously described method [70] with minor modification. For H_2_O_2_ localization, stained leaves were placed in vacuum along with 0.5 mg·mL^− 1^ fresh DAB solution prepared by 25 mM Tris-HCl (pH 3.8) and kept for 12 h at room temperature. Brown spots appeared on the surface of leaves because of the reaction between DAB and H_2_O_2_. For O_2_^•−^ detection, the other leaf samples were immersed with 1 mg·mL^− 1^ NBT solution, which was made with 10 mM phosphate buffer (pH 7.8), and incubated at room temperature in the dark for 12 h. Blue spots were also present on leaves because of the reaction of NBT and O_2_^•−^. Both of the stained leaf samples were bleached by boiling in 95% ethanol for 20 min to remove chlorophyll. Then, the samples were placed into absolute ethanol for several hours before taking photos with a digital camera.

### Determination of H_2_O_2_ production level

The H_2_O_2_ concentration in leaves was estimated by slightly modifying a method described by Velikova et al. [71]. First, 0.2 g leaves were homogenized with 1.6 mL 0.1% trichloroacetic acid (TCA) in an ice bath for 30 min and centrifuged at 12000×*g* for 20 min at 4 °C. Then, 0.5 mL 0.1 M potassium phosphate buffer (pH 7.8) and 1 mL 1 M KI (Potassium Iodine) were added to 0.5 mL supernatant and kept in a dark place for 1 h. The absorbance was measured at 390 nm. Finally, the H_2_O_2_ content was quantified with a standard curve and expressed as μmol g^− 1^ FW.

### Determination of O_2_^•−^ production rate

The O_2_^•−^ generation rate was determined following the procedure reported by Nahar et al. [72] with some alterations. Briefly, 0.2 g leaves were homogenized with 2 mL 50 mM phosphate buffer (pH 7.8) and centrifuged at 12000×*g* for 20 min at 4 °C. Then, 0.5 mL 50 mM phosphate buffer (pH 7.8) and 0.1 mL 10 mM hydroxylamine hydrochloride were mixed in 0.5 mL supernatant and incubated at room temperature for 30 min. After incubation, 1 mL 17 mM sulfanilamide and 1 mL of 7 mM naphthylamine were added to the mixture and incubated for 30 min. The absorbance reading of the mixture was measured at 530 nm. O_2_^•−^ production was then calculated with a standard curve of NaNO_2_ and expressed as nmol g^− 1^ min^− 1^ FW.

### Membrane injury index (MII) measurement

Membrane injury index (MII) of leaves was computed to the method outlined by Jahan et al. [10] with few modifications. Briefly, 0.5 g fresh leaves were thoroughly washed with deionized water, cut into small pieces, put into tubes filled with 20 mL deionized water, and placed at room temperature for 4–5 h under dark conditions in a shaker; then, the initial electrical conductivity (EC1) in the bathing solution was determined by a portable conductivity meter (DDS-307, Shanghai Precision and Scientific Instrument LTD., Shanghai, China). Subsequently, the samples were boiled at 95 °C for 20 min and cooled to room temperature, and the final electrical conductivity (EC2) was measured in the bathing solution. Simultaneously, we determined the deionized water conductivity (EC0). The MII was calculated as follows:

$$\mathrm{MII}\ \left(\%\right)=\frac{\mathrm{EC}1-\mathrm{EC}0}{\mathrm{EC}2-\mathrm{EC}0}\times 100$$

### Lipid peroxidation measurement

Lipid peroxidation was inferred based on MDA content in leaves, which was measured as described by Alexieva et al. [73] with slight adjustments. First, 0.2 g leaf samples were homogenized in a 1.6 mL 0.1% (w/v) TCA solution and centrifuged at 4 °C for 20 min at 12000×*g*. From the supernatant, a 1.0-mL aliquot was added to 1.0 mL TCA containing 0.67% TBA; then, the sample was boiled at 95 °C for 15 min and kept on ice for cooling. Subsequently, the mixture was centrifuged at 4400×*g* for 10 min. Then, MDA content was measured at 532 nm and 600 nm by a spectrophotometer (Evolution 300, Thermo Fisher Scientific, Waltham, MA, USA).

### Proline content determination

The proline content was evaluated following the method described by Bates et al. [74]. Fresh leaf samples (0.2 g) were digested in 3% sulphosalicylic acid followed by centrifugation at 12000×*g* for 20 min at 4 °C. The same amount of glacial acetic acid and ninhydrin solutions were incorporated in the supernatant and incubated for 30 min. Consequently, the sample was heated at 100 °C for 1 h, and 5 mL toluene was added after cooling. Toluene absorbance was read at 520 nm by a spectrophotometer (Spectronic 20D, Milton Roy, Philadelphia, PA, USA).

### Relative water content measurement

The relative water content (RWC) was calculated using the method established by Barrs and Weatherley [75] with some changes. Fully developed leaves were arbitrarily detached from treated plants and immediately weighed as FW, followed by soaking in distilled water and incubation for 6 h at room temperature. Then, the excess surface water was removed with a paper towel, and the turgid weight (TW) was recorded. Leaf samples were then oven dried at 80 °C for 72 h to obtain the dry weight (DW). RWC was calculated using the following equation:

$$\mathrm{RWC}\ \left(\%\right)=\frac{\mathrm{FW}-\mathrm{DW}}{\mathrm{TW}-\mathrm{DW}}\times 100$$

### Leaf enzymes activity assays

Fresh leaf samples (0.2 g) were digested with a chilled pestle and mortar in 1.6 mL 50 mM pre-cooled phosphate buffer (pH 7.8), and supernatants were obtained by centrifugation of the homogenate at 12000×*g* for 20 min at 4 °C. The supernatants were then used to estimate the antioxidant enzymes activities.

SOD activity (EC 1.15.1.1) was calculated using a modified version of the protocol described by Giannopolitis and Ries [76], and Maresca et al. [77] described the procedure that was used to estimate POD (EC 1.11.1.7) activity. Briefly, a 40-μL enzyme extract was added to a 3-mL reaction mixture that contained 14.5 mM Met, 30 μM EDTA–Na_2_ solution, 50 mM phosphate buffer (pH 7.8), 2.25 mM NBT solution, and 60 μM riboflavin solution. The SOD content was monitored at 560 nm. For POD activity assay, 40 μL enzyme solution was mixed with a 3-mL reaction volume that included 0.2 M phosphate buffer (pH 6.0), 50 mM guaiacol, and 2% H_2_O_2_ solution, and absorbance was quantified at 470 nm.

To determine catalase (CAT, EC 1.11.1.6) activity, the protocol described by Dhindsa et al. [78] was used. Briefly, a 0.1 mL enzyme solution was added followed by a 3-mL reaction mixture that contained 0.15 M phosphate buffer (pH 7.0) and 0.3% H_2_O_2_ solution. The level of activity was calculated at 240 nm.

To examine APX (EC 1.11.1.11) activity, the method described by Nakano and Asada [79] was used. Briefly, a 1.6-mL assay mixture that consisted of 50 mM phosphate buffer (pH 7.0), 0.1 mM EDTA–Na_2_, 5 mM AsA, and 20 mM H_2_O_2_ were added to 0.1 mL enzyme solution. The activity was calculated at 290 nm.

AsA content was quantified as previously reported by Logan et al. [80]. Briefly, 0.1 g leaf samples were homogenized in 1.5 mL 6% pre-chilled HClO_4._ After grinding, the sample was centrifuged at 12000×*g* for 15 min at 4 °C, and supernatant was collected for further analysis. For neutralization, 200 mM sodium acetate buffer (pH 5.6) was added to the supernatant and AsA was assayed at 265 nm; the absorbance reading was recorded before and after incubation of the supernatant in 1.5 units of AsA oxidase for 15 min.

GSH was assayed with the protocol priorly described by Griffith [81]. Briefly, the leaf sample (0.1 g) was ground in 1.5 mL 5% sulfosalicylic acid, and the homogenized sample was centrifuged at 4 °C for 20 min at 12000×*g*. The supernatant was neutralized with 200 mM sodium acetate buffer (pH 5.6). Then, 5, 5-dithiobis-(2-nitrobenzoic acid) was incorporated for enzymatic recycling of GSH. The GSH content was calculated by recording the absorbance at 412 nm with a spectrometer.

MDHAR (EC 1.6.5.4) activity was determined based on the change (due to NADPH oxidation) in absorbance at 340 nm, as previously described by Hossain et al. [82].

DHAR (EC 1.8.5.1) activity was assayed based on the change in absorbance at 265 nm, as described by Nakano and Asada [79].

Glutathione S-transferase (GST) activity was assayed with GST detection kit (Solarbio Life Science, Beijing, China) following the manufacturer’s instructions. First, fresh leaves (0.1 g) were ground with extraction buffer (1 mL) in an ice bath and homogenized by centrifugation at 4 °C for 10 min at 8000×*g* with the supernatant used for testing GST. GST activity was calculated using the molar extinction coefficient 9.6 × 10^3^ Lmol^− 1^ cm^− 1^.

Glutathione reductase (GR) activity was determined with GR detection kit (Solarbio Life Science, Beijing, China) following the manufacturer’s instructions. Briefly, 0.1 g leaf tissue was taken and homogenized in 1 mL extraction solution in an ice bath and centrifuged at 10000×*g* for 10 min at 4 °C, and the supernatant was used to determine GR activity. To calculate GR, 6.22 × 10^3^ L mol^− 1^ cm^− 1^ was used as the extinction coefficient.

Nitrate reductase (NR) activity was measured with NR detection kit (Solarbio Life Science, Beijing, China) following the manufacturer’s instructions. First, 0.1 g fresh leaf samples were gently washed and the water was removed from the leaf surface. Samples were incubated for 2 h in work solution under dark condition in room temperature and then kept at − 20 °C for 30 min. Then, samples were taken, ground in an ice bath with 1 mL extract solution, and centrifuged at 4000×*g* for 10 min; the supernatant was used to determine NR activity.

Nitric oxide synthase (NOS) activity was assayed by NOS detection kit (Solarbio Life Science, Beijing, China) following the manufacturer’s instructions.

NO content was quantified using NO detection kit (Solarbio Life Science, Beijing, China) according to the manufacturer’s protocol.

### Protein extraction

The protein content was determined using Bovine serum albumin (BSA) as the standard following the method described by Bradford [83].

### Determination of endogenous free polyamines

Endogenous free polyamines content were assayed as the approaches reported by Shen et al. [84] with minor modifications. Briefly, 0.5 g leaf tissue was homogenized in 5% (v/v) cold perchloric acid and incubated on ice for 1 h. Then, homogenates were centrifuged for 20 min at 12000×*g* and the upper supernatant was used to determine the free PAs. A 0.7 mL aliquot was reacted with 1.4 mL NaOH (2 N) and 15 μL benzoyl chloride, and then gently vortexed the mixer and incubated for 30 min at 37 °C. Later, to stop the reaction, 2 mL saturated NaCl was added to the solution. To extract benzoyl PAs, 2 mL cold diethyl ether was mixed into the solution, which was then centrifuged at 3000×*g* for 5 min. The extracted benzoyl PAs were evaporated to dryness and then re-dissolved in 1 mL of 64% (v/v) methanol. To separate and analyze the PAs content, we used UHPLC (Ultimate 3000, Thermo Scientific, San Jose, CA, USA) with a C18 reversed-phase column at a flow rate of 0.8 mL min^− 1^.

### Total RNA extraction and quantitative real-time PCR analysis

Total RNA was extracted from 0.1 g tomato leaves tissues using the RNAsimple Total RNA Kit (TIANGEN, Beijing, China) according to the manufacturer’s instructions. One microgram of total RNA was reverse-transcribed into cDNA using a SuperScript First-strand Synthesis System for quantitative real-time PCR based on the manufacturer’s instructions (Takara, Tokyo, Japan). The gene-specific primers were designed using DNA sequences from the NCBI database (https://www.ncbi.nlm.nih.gov/), and Sol Genomics Network (solgenomics.net) and the primer pair sequences are listed in Additional file 1: Table S1. Real-time PCR was performed on a StepOnePlus™ Real-Time PCR System (Applied Biosystems, Foster City, CA, USA) with ChamQ Universal SYBR qPCR Master Mix (Vazyme Biotech Co., Ltd., Nanjing, China). The total reaction system volume was 20 μL, which consisted of 10 μL ChamQ SYBR qPCR Master Mix (2×), 0.4 μL ROX reference dye 1 (50×), 2 μL template cDNA (10×), 0.8 μL each specific primer (10 μM), and 6 μL sterilized ddH_2_O. Three biological replicates were performed for each reaction and the cycling conditions were as follows: 95 °C for 5 min, followed by 40 cycles of denaturation at 95 °C for 15 s and annealing at 60 °C for 1 min, and a final extension at 95 °C for 15 s. Relative expression was calculated using the 2^−ΔΔCt^ formula [85], and the mRNA expression level was normalized against actin (used as an internal control) and compared.

### Statistical analysis

At least five independent biological replicates were performed for each treatment, and three replicates were performed for the whole experiment. All of the data were statistically analyzed with SPSS 20.0 (SPSS Inc., Chicago, IL, USA). One way analysis of variance was performed, and statistically significant differences among the treatments were determined using Tukey’s honest significant difference test at *P* < 0.05. A transcript expression heatmap was created using the TBtools statistic package. Origin Pro 8.0 was used to make graphs.

## Additional files

Additional file 1: **Table S1.** List of primers used for qRT-PCR assays. (DOCX 29 kb)

## Data Availability

The datasets generated and analyzed during the current study are available from the corresponding author on reasonable request.

## Abbreviations

_1_O^2^: Singlet oxygen
ADC: Arginine decarboxylase
APX: Ascorbate peroxidase
AsA: Ascorbate
BSA: Bovine serum albumin
CAT: Catalase
DHAR: Dehydroascorbate
DNA: Deoxy ribo nucleic acid
EC: Electrical conductivity
GR: Glutathione reductase
GSH: Glutathione
GST: Glutathione S-transferase
H_2_O_2_: Hydrogen peroxide
HsfA2: Heat shock transcription factors A2
HSP90: Heat shock protein 90
HSPs: Heat shock proteins
MDA: Malondialdehyde
MDHAR: Monodehydroascorbate reductase
MII: Membrane injury index
NO: Nitric oxide
NOS: Nitric oxide synthase
NR: Nitrate reductase
O_2_^•−^: Superoxide anion
ODC: Ornithine decarboxylase
OH^•^: Hydroxyl radical
P5CS: Delta 1-pyrroline-5-carboxylate synthetase
PAO: Polyamine oxidase
PAs: Polyamines
POD: Peroxidase
PUT: Putrescine
RBOH: Respiratory burst oxidase
RNA: Ribo nucleic acid
ROS: Reactive oxygen species
RWC: Relative water content
SAMDC: S-adenosyl methionine decarboxylase
SOD: Superoxide dismutase
SPD: Spermidine
SPDS: Spermidine synthase
SPM: Spermine
SPMS: Spermine synthase
