# Supplementary material for: Melatonin alleviates heat-induced damage of tomato seedlings by balancing redox homeostasis and modulating polyamine and nitric oxide biosynthesis
Source: BMC Plant Biol. 2019 Oct 7;19:414. doi: 10.1186/s12870-019-1992-7 (PMC6781414; doi:10.1186/s12870-019-1992-7)
Supplement: Supplementary file 1 — Table S1. List of primers used for qRT-PCR assays. (DOCX 29 kb) [file 12870_2019_1992_MOESM1_ESM.docx]

| **Gene full name** | **Gene acronym** | **Accession number** | **Forward primer (5^ˊ^-3^ˊ^)** | **Reverse primer (5^ˊ^-3^ˊ^)** |
| --- | --- | --- | --- | --- |
| Superoxide dismutase | *SOD* | Solyc02g082590 | ACTACTCCCAGTTGCATCCC | CACCAGGAGCAGCCATGATA |
| Catalase | *CAT* | Solyc12g094620 | GCAGCTCCCAGTTAATGCTC | AGCAGGACGACAAGGATCAA |
| Peroxidase | *POD* | Solyc07g052510 | GGTCTGTTCCAATCCGATGC | CACCAGCACTCCCTGTCTTA |
| Ascorbate peroxidase | *APX* | Solyc06g005150 | GGCACTCTGCTGGTACCTAT | GGAGAGAGTGGGAAACTGCT |
| Glutathione reductase | *GR* | Solyc09g065900 | GGAGCCATAGAGGTTGACGA | CTCCTCCCTCCATCAAAGCA |
| Glutathione S-transferase | *GST* | Solyc05g006740 | TTGTCTGGTGCTCTCTTCGT | TGAGAGCAACTGGAGCATGA |
| Monodehydroascorbate reductase | *MDHAR* | Solyc08g081530 | CGGACAGTTCCGAACAAACA | CCCGTGCAATTCGGTTGTAT |
| Dehydroascorbate reductase | *DHAR* | Solyc05g054760 | GAGGTGAACCCTGAAGGGAA | CCCACAGAGGCAAATTCAGG |
| Heat shock protein 90 | *HSP90* | Solyc05g010670 | GCAATGGCTCTTGGAGGAAG | CGGTCCTTACTCAGATGGCT |
| Respiratory burst oxidase | *RBOH* | Solyc08g081690 | TAACAGCTAAGGGTGCTGCT | CAAGGTGATTACCGGCATGG |
| Heat shock factor A 2 | *HsfA2* | Solyc08g062960 | CCCAGTGCTACAGGAAGTGA | TCATCAGCCACTGGTTCCAT |
| Nitrate reductase | *NR* | Solyc11g013810 | CAGTGGCTCCTCCAACACTA | CAAGAAACGTGAGGCATCGT |
| Nitric Oxide Reductase | *NOS* | Solyc03g093240 | AGAGACCTACCTGCGATGTG | GTACCACTGTCCTCCTGCTT |
| Delta 1-pyrroline-5-carboxylate synthetase | *P5CS* | Solyc08g043170 | CATAACCAGTGGCTTTGCGA | GAAGACGTCTGGAACATGCC |
| Arginine decarboxylase 1 | *ADC1* | Solyc01g110440 | CATCCAGTGATTTGCAGCGA | GTAAACCACCCGAAGATGGC |
| Arginine decarboxylase 2 | *ADC2* | Solyc10g054440 | GTCGGATATGGCCTTCAGGA | CTTCACGTTCTTCCGGTCAC |
| Ornithine decarboxylase 1 | *ODC1* | Solyc04g082030 | GCTCAACTCGGAATGCCAAA | CTCAGGGAAGTCGTGGAAGT |
| Ornithine decarboxylase 2 | *ODC2* | Solyc03g098300 | GGAGCATTGCCGGAAGAAAT | TCGCTTGGCGATAAATGGTG |
| Sprmine synthease | *SPMS* | Solyc03g007240 | GCAGGGAGTGGAGTCAAGAT | TGCTATGCGTGTGACAAGTG |
| S-adenosylmethionine decarboxylase 1 | *SAMDC1* | Solyc05g010420 | CCGAGTCTAGCCTCTTCGTT | AAATGAAGCTCCCACGGGTA |
| S-adenosylmethionine decarboxylase 2 | *SAMDC2* | Solyc02g089610 | AAACTCTTATGGCCCTGGCT | AGGCTGGACTCTGAAAGGAC |
| Spermidine synthase 1 | *SPDS1* | Solyc04g026030 | AACTCACAGAGCGGGATGAA | AAGAATGCCGAGACACCTCA |
| Spermidine synthase 2 | *SPDS2* | Solyc05g005710 | TATCTACGCAGGCTGAGAGC | AGGTCCCTCAGTAGAGCAGA |
| Spermidine synthase 3 | *SPDS3* | Solyc08g014310 | GCCATCACAATCACCACCAA | ACCTGGCCATAACGCACTAA |
| Spermidine synthase 4 | *SPDS4* | Solyc06g053510 | GCAACTCCCACACCCATAAC | ACCTTCCCATACGTTGCTGA |
| Spermidine synthase 5 | *SPDS5* | Solyc06g053520 | TGATCCACGTGCAACACTTT | GAGTAAACTCCTCCTGGCCTT |
| Polyamine oxidase 1 | *PAO1* | Solyc01g087590 | CCTCTGTGATCATCGTCGGA | CTACTCCGCCGAATTCCTCT |
| Polyamine oxidase 2 | *PAO2* | Solyc05g018880 | TGCAGGAATGGCAGGTCTTA | TGGATCCAAGTAGCACCCAT |
| Actin |  | Solyc03g078400 | TGGTCGGAATGGGACAGAAG | CTCAGTCAGGAGAACAGGGT |

**Table S1** List of primers used for qRT-PCR assays
